# Draft Genome Sequence of *Rhodococcus opacus* Strain M213 Shows a Diverse Catabolic Potential

**DOI:** 10.1128/genomeA.00144-12

**Published:** 2013-02-14

**Authors:** Ashish Pathak, Stefan J. Green, Andrew Ogram, Ashvini Chauhan

**Affiliations:** a School of the Environment, Florida A&M University, Tallahassee, Florida, USA; b DNA Services Facility, University of Illinois at Chicago, Chicago, Illinois, USA; c Department of Biological Sciences, University of Illinois at Chicago, Chicago, Illinois, USA; d Soil and Water Science Department, University of Florida, Gainesville, Florida, USA

## Abstract

Soil-borne Gram-positive bacteria from the genus *Rhodococcus* metabolize a range of aromatic hydrocarbons and also produce a variety of value-added products, such as triacylglycerols and steroids. We report the draft genome sequence of *Rhodococcus opacus* strain M213 (9,193,504 bp with a G+C content of 66.99%), providing a comprehensive understanding of the repertoire of metabolic genes of this strain.

## GENOME ANNOUNCEMENT

Much of what is known about the biochemistry and genetics of naphthalene (NAP) metabolism is based on *Pseudomonas putida* G7 and related gammaproteobacteria (1, 2). The catabolic pathways of soil *Actinobacteria*, such as the rhodococci, are not homologous to those of the pseudomonads and hence remain poorly understood (3–5). We are interested in NAP degradation by *Rhodococcus opacus* strain M213, which was isolated from a fuel oil-contaminated soil sample (6). Previously described NAP degradative pathways generate salicylate (SAL) as a metabolic intermediate (1, 2, 7). Several lines of evidence suggest that *R. opacus* M213 encodes an alternate pathway, in which *o*-phthalate is generated as a key metabolic intermediate during growth on NAP (6, 8).

To understand fully the metabolic potential of strain M213, genomic DNA was prepared for shotgun sequencing using the Nextera kit (Epicenter, Madison, WI), with size selection (400- to 800-bp fragments) performed using a Pippin Prep automated electrophoresis instrument (Sage Scientific, Beverly, MA) and sequenced using 100-base paired-end sequencing on an Illumina HiSeq 2000 system. Approximately 87 M reads were generated in pairs and assembled by the *de novo* assembler within the software package CLC Genomics Workbench v5.0 (CLCbio, Cambridge, MA). A total of 483 contigs of length ≥200 bases were generated, with a sum of ~9.2 Mb, an N_50_ of 79,111 bases, and an average coverage of >800×. Note that 95% of the sequence data assembled were present in the 158 largest contigs (N_95_, 9.4 kb), and a total of 350 contigs of ≥500 bases were assembled (99.55% of total assembly). Contigs were successfully used for annotation and gene prediction by Integrated Microbial Genomes (IMG) Expert Review ER (9) using Prodigal (10), which compares the translated proteins with the nonredundant proteins database (NR) at GenBank, Pfam (11), TIGRFam (12), InterPro (13), Kyoto Encyclopedia of Genes and Genomes (KEGG) (12) and Clusters of Orthologous Groups (COG) (14) databases using BLASTp and HMMER. The genome was also analyzed by Rapid Annotations using Subsystems Technology (RAST) (15).

Heat map analysis and principal components analysis (PCA) using IMG ER showed that M213 significantly differs from the other eight rhodococci for which genome sequences are available. Strain M213 contains a total of 8,942 putative genes, with 75.14% of genes associated with protein-coding functions, whereas 24.06% genes have unknown functions. Approximately 22% of the protein-coding genes were connected to KEGG pathways, with 401 genes involved in the metabolism of polycyclic aromatic hydrocarbons (PAHs) (naphthalene, phenanthrene, anthracene, and benzo[a]pyrene) and an array of halogenated aromatics and aromatic hydrocarbons, including pesticides (dichlorodiphenyltrichloroethane [DDT] and atrazine). With respect to NAP, genes similar to those involved in the oxidation of both salicylate and *o*-phthalate were detected, suggesting the possibility of dual pathways for NAP degradation in strain M213. Also present were 284 genes for the biosynthesis of terpenoids, polyketides, and other secondary metabolites, such as caffeine, flavonoids, indole, isoquinoline, and alkaloids, along with 300 genes associated with unsaturated and saturated fatty acid biosynthesis and metabolism; this makes strain M213 a lucrative candidate for the industrial production of biofuel precursors and steroids.

### Nucleotide sequence accession numbers.

This Whole Genome Shotgun project has been deposited at DDBJ/EMBL/GenBank under the accession no. AJYC00000000. The version described in this article is the second version, AJYC02000000.
